# Peg-Shaped Lateral Incisors Treated with Ceramic Three-Quarter Crown

**DOI:** 10.1155/2021/9412638

**Published:** 2021-12-29

**Authors:** Rim Kallala, Sarra Nasri, Amani Adli, Rihab Dakhli, Chaouch Mohamed Habib, Touzi Soumaya, Nouira Zohra, Belhassen Harzallah, Mounir Cherif

**Affiliations:** ^1^Department of Dental Anatomy at the Faculty of Dental Medicine Monastir, Tunisia; ^2^University of Monastir, Faculty of Dental Medicine, Research Laboratory of Occlusodontics and Ceramic Prostheses LR16ES15, 5000 Monastir, Tunisia; ^3^Department of Fixed Prosthodontics of the Dental Clinic of Monastir, Tunisia; ^4^Dental Faculty of Monastir, Tunisia

## Abstract

In recent years, patients' expectations are becoming higher in terms of esthetic dentistry. Having the ideal smile is becoming a purpose as the appearance of stars and famous persons have been undoubtedly improved through smile correction. Peg-shaped lateral incisors are a common dental form aberration which could distort the smile and may hamper patient psychology because of the smaller shape and size in disharmony with other teeth. Three-quarter ceramic veneers are a mini-invasive approach which could solve the esthetic problem of peg-shaped teeth and ensure patient's satisfaction. The present paper was about a clinical case with peg-shaped lateral incisors treated with three-quarter ceramic crowns. It would also highlight the preparation particularities.

## 1. Introduction

In recent years, esthetic requirements are highly increasing in dentistry for both anterior and posterior teeth [1]. Having the ideal smile is becoming a purpose as the appearance of stars and famous persons have been undoubtedly improved through smile correction [2].

This smile enhancement concerns especially central and lateral incisors as they are the most involved teeth in smile attractiveness [3–5]. Peg-shaped lateral incisors are a common dental form aberration which could distort the smile and may hamper patient psychology because of the smaller shape and size in disharmony with other teeth [6]. The management of peg-shaped lateral incisors has to be carefully performed. An attentive communication with the patient is necessary to ensure his satisfaction. This article is aimed at presenting a clinical case of peg-shaped lateral incisors treated with a ceramic three-quarter crown. It would also highlight the preparation particularities.

## 2. Clinical Presentation

**Figure 1:**
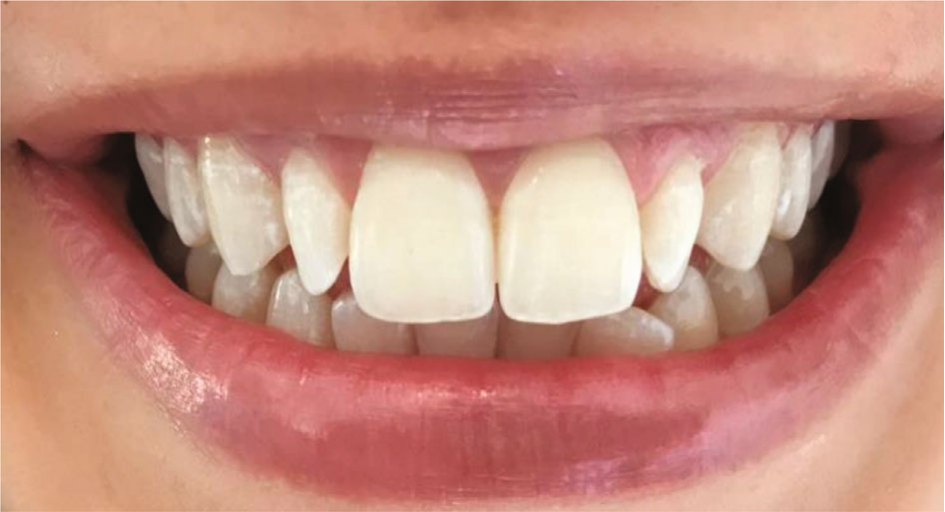
Initial situation showing peg-shaped lateral incisors.

It was about a 20-year-old healthy dental student who consulted the prosthetic department of the clinical dentistry of Monastir Tunisia. She complained from her ugly smile (Figure 1). The patient had high esthetic expectations. The clinical examination showed long peg-shaped laterals which were undersized with a conic shape. An alginate (Lascod, Firenze, Italy) impression was taken.

**Figure 2:**
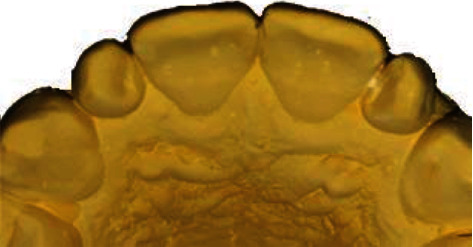
Rotation of both lateral incisors on diagnostic cast.

**Figure 3:**
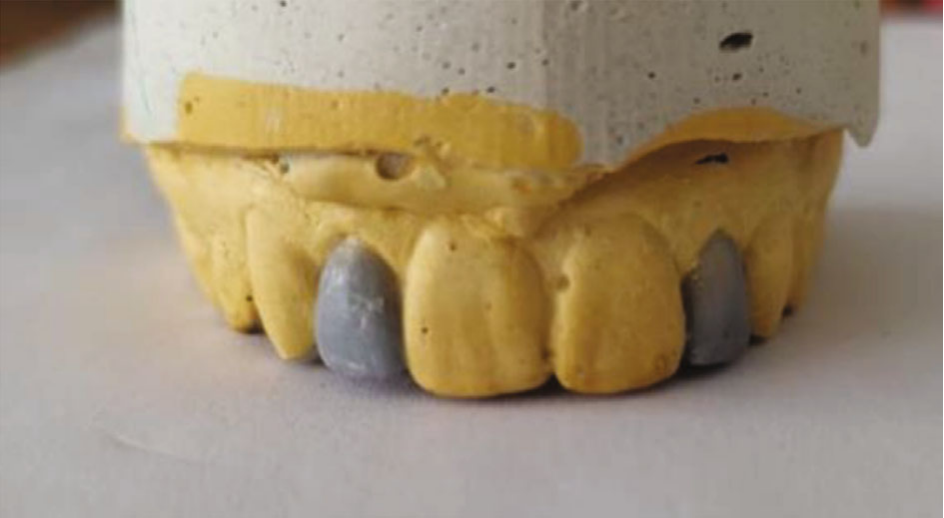
Wax-up.

On the diagnostic cast, these teeth presented also a distolingual rotation, especially for the 22 teeth (Figure 2). Taking into consideration the teeth version and vitality, a ceramic three-quarter crown was indicated. The wax-up was done, and the mock-up tested the final outcome which satisfied the patient (Figure 3).

**Figure 4:**
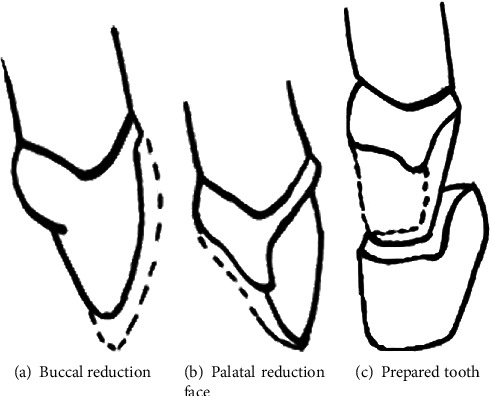
Schematic preparation design.

The preparation of the three-quarter crown design (Figure 4) was similar to veneer preparation with incisal overlap. The retractor cord (Ultradent, South Jordan, Utah, USA) was placed for gingival displacement. A mock-up driven technique preparation restricted to enamel tissue was done. With diamond-rounded bur, grooves were created in order to limit the preparation depth. The labial surface was then prepared with a round-end tapered bur without breaking the contact point. The incisal reduction was 2 mm.

Atypical preparation was done for the palatal face: it was a slopping plan correcting the tooth version. The finish line was incisal to the collar. The objective was to exclude the dentoprosthetic joint from occlusal contacts.

**Figure 5:**
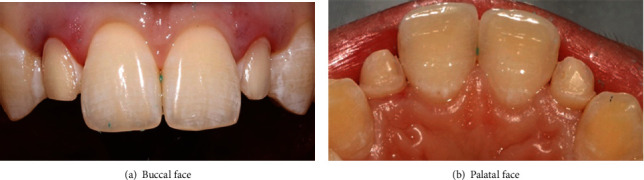
Preparation design.

Once the preparation was validated (Figure 5), an impression using polydimethylsiloxane material (Mono Ghenesyl, Lascod, Firenze, Italy) was taken and sent to the laboratory where the master cast was scanned.

**Figure 6:**
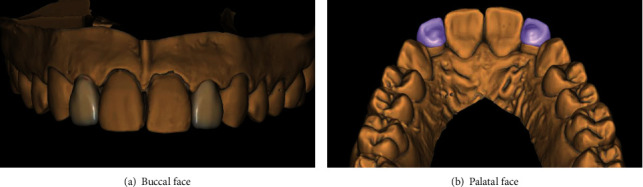
Virtual three-quarter crowns conceptioned by Exocad Software 3.00.

**Figure 7:**
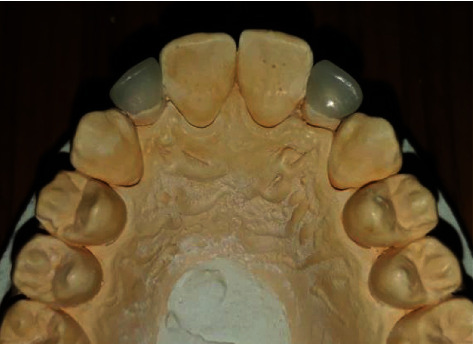
Milled three-quarter crowns.

**Figure 8:**
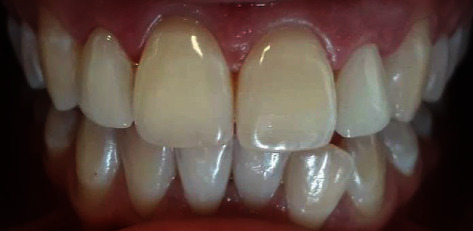
Intraoral checking of the three-quarter crowns.

Prosthetic pieces were confectioned using exocad Dental 3.00 Galway software (Fraunhofer Society, Germany) (Figure 6) and milled. When the prosthetic pieces were received (Figure 7), marginal fit was checked intraorally (Figure 8), and then, morphological characteristics were optimized. Occlusal contacts were also verified: with static occlusion, only tight contacts were left, and during mandible movements, these teeth were completely excluded. Only the central incisor supported the propulsion movement.

**Figure 9:**
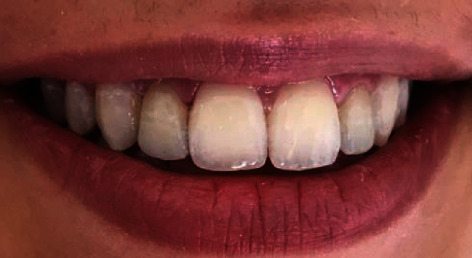
Final esthetic result which shows the natural aspect of the restoration.

The bonding was the last step: a retractor cord was placed in order to optimize the bonding quality. The bonding system used was Variolink II (Ivoclar Vivadent, Schaan, Liechtenstein). The outer surface of the prosthetic pieces was protected by polydimethylsiloxane and then carefully treated by hydrofluoric acid and silinated. Intraorally, the prepared teeth were etched with phosphoric acid 37% for 30 seconds, then rinsed and air dried. An adhesive was carefully applied. After that, after the resin bonding application, they were correctly placed. Excess were removed, and the tooth was light cured for 30 s from facial and palatal surfaces (D Lux®, Diadent Group International, Chungcheongbuk-do, South Korea). All clinical procedures of the bonding are listed in Table 1. The goal was achieved, and the patient was extremely satisfied especially with the natural aspect of the outcome (Figure 9).

## 3. Discussion

For peg-shaped lateral incisors, many therapeutic treatments could be indicated: full crowns, veneers, and direct or indirect composite resins [6]. Good physical properties, esthetic quality, and marginal integrity make the composite resin a reliable material for such a situation [7–9]. According to Mittal et al. [6, 10], the hybrid composite could offer good results. Thus, optical proprieties of vitroceramic would optimize the esthetic outcome, especially when concerned teeth are not discolored. Besides, ceramic is characterized by the color stability [7] and better resistance to abrasion compared to resin [7].

In the presented case, ceramic full crowns could be indicated. But, as the patient was young, it would be invasive towards the pulp vitality because of the tooth version. A treatment alternative could have been a prefabricated veneer [11] as it is a minimal invasive approach which performs the shape and color. Some situations do not require preparation. Thus, this therapeutic option has to be carefully indicated to ensure its longevity [12]. Tooth vitality, color, position, and occlusion must been well studied in order to raise the most suitable therapeutic indication [13]. However, for important malpositions as described in the presented case, ceramic veneers could not be indicated because of the excessive preparation needed which will expose the dentin tissue [13]. Consequently, the bonding is worse and could lead to failure. Authors reported that observed veneers bonded to dentin were approximately ten times more at risk to fail comparing to those bonded to enamel [14]. Otherwise, because of the teeth rotation, the palatal preparation for ceramic veneers could not offer a suitable seat for the cosmetic material which led to placing the palatal finish line incisally. Also, that preparation had to be out of the occlusal contact zones; it was necessary to further place it near to the collar. For all cited reasons, the ceramic three-quarter crown was the most minimally invasive solution which allowed the tooth shape correction while respecting the pulp vitality [15].

Also, the palatal surface would provide important enamel surface for better bonding. According to El-Sherif and Jacobi [16], the three-quarter crown offers greater resistance and retention compared to ceramic veneers. It provides also more natural tooth contours. Very few clinical cases were published in the literature of the three-quarter crown for anterior teeth. El-Sherif and Jacobi [16] explained the preparation particularities of this restoration. They proposed 1.5 mm of reduction in the incisal edge. In the presented case, the reduction was 2 mm because the teeth were as long as central incisors. Consequently, the reduction allowed 1 mm for the ceramic thickness and 1 mm ensuring shorter lateral incisors. They suggested also that the palatal finish line should be 1.5 mm gingival to the incisal edge. In the presented case, it was placed 3 mm incisal to the collar. They have insisted on the fact that it should be placed 1 mm gingival or incisal of centric contacts area which was respected.

Limits of the present clinical presentation are those of ceramic veneers in relationship of the restoration dislodgement. The bonding procedure has to be carefully performed. The patient was informed concerning this possibility. That's why, she was adviced to avoid incising hard food using her anterior teeth. Later, this restoration could be replaced by a full-covering crown until pulp tissue would be retracted due to aging. Consequently, the pulp vitality would be respected.

## 4. Conclusion

The three-quarter crown is a reliable esthetic solution for peg-shaped lateral incisors, which have all the advantages of ceramic veneers. Its indication should be carefully raised taking into consideration various factors. The preparation design is crucial to ensure longevity of such restoration. The balance is between the sufficient preparation for the material thickness and the enamel preservation. The wax-up and mock-up are important to raise the best therapeutic indication for each clinical situation.

## Figures and Tables

**Table 1:** The bonding procedure steps (Ivoclar Vivadent, Schaan, Liechtenstein).

Prosthetic pieces		Tooth surface
Fluhidroque acid	Surface treatment	Phosphoric acid 37%
Silinated		Adhesive application
Rinsed and air dried		Rinsed and air dried
Resin bonding application		
Consolidation		
Excess removing		
Lightning for 30 s (D Lux®, Diadent Group International, Chungcheongbuk-do, South Korea)		

## References

[1] Kallala R., Chaouch M. H., Nasr K., Courset T. (2011). Step-by-Step Esthetic Rehabilitation with Chairside System. *Case Reports in Dentistry*.

[2] Rehman K., Khan F. R., Rahman M. (2011). Assessing the perception of smile attractiveness in young adults. *Journal of the Pakistan Dental Association*.

[3] Sriphadungporn C., Chamnannidiadha N. (2017). Perception of smile esthetics by laypeople of different ages. *Progress in Orthodontics*.

[4] Machado A. W. (2014). 10 commandments of smile esthetics. *Dental Press Journal of Orthodontics*.

[5] Sarver D. M. (2001). The importance of incisor positioning in the esthetic smile: the smile arc. *American Journal of Orthodontics and Dentofacial Orthopedics*.

[6] Mittal N., Mohandas A. (2018). Management of peg-shaped lateral with new minimal invasive restorative technique-componeer: a case report. *Journal of Dental Advance*.

[7] Saatwika L., Anuradha B., Prakash V., Anuradha B., Subbiya A. (2019). Esthetic correction of Anteriors: a case report. *European Journal of Molecular & Clinical Medicine*.

[8] Migne P., Belser U. C. (2003). Porcelain versus composite inlays/onlays: effects of mechanical loads on stress distribution, adhesion and crown flexure. *The International Journal of Periodontics & Restorative Dentistry*.

[9] Nakamura T., Imanishi A., Kashima H., Ohyama T., Ishigaki S. (2001). Stress analysis of metal-free polymer crowns using the three-dimensional finite element method. *The International Journal of Prosthodontics*.

[10] Conrad H. J., Seong W. J., Pesun I. J. (2007). Current ceramic materials and systems with clinical recommendations: a systematic review. *The Journal of Prosthetic Dentistry*.

[11] Novelli C., Pascadopoli M., Scribante A. (2021). Restorative treatment of amelogenesis imperfecta with prefabricated composite veneers. *Case Reports in Dentistry*.

[12] Chai S. Y., Bennani V., Aarts J. M., Lyons K. (2018). Incisal preparation design for ceramic veneers: a critical review. *The Journal of the American Dental Association*.

[13] Kallala R., Salhi Daly M., Gassara Y. (2021). Rationalizing indication of ceramic veneers: a systematic review. *EAS Journal of Dentistry and Oral Medicine*.

[14] Farias-Neto A., de Medeiros F. C. D., Vilanova L., Simonetti Chaves M., Batista F., de Araújo J. J. (2019). Tooth preparation for ceramic veneers: when less is more. *International Journal of Esthetic Dentistry*.

[15] Golberg M. B., Siegel S. C., Rezakani N. (2013). Unique CAD/CAM three-quarter crown restoration of a central incisor: a case report. *General Dentistry*.

[16] El-Sherif M., Jacobi R. (1989). The ceramic reverse three-quarter crown for anterior teeth: Preparation design. *The Journal of Prosthetic Dentistry*.

